# Degradability of Polyurethanes and Their Blends with Polylactide, Chitosan and Starch

**DOI:** 10.3390/polym13081202

**Published:** 2021-04-08

**Authors:** Joanna Brzeska, Agnieszka Tercjak, Wanda Sikorska, Barbara Mendrek, Marek Kowalczuk, Maria Rutkowska

**Affiliations:** 1 Department of Industrial Product Quality and Chemistry, Gdynia Maritime University, 83 Morska Street, 81-225 Gdynia, Poland; m.rutkowska@wpit.umg.edu.pl; 2 Group ‘Materials+Technologies’ (GMT), Department of Chemical and Environmental Engineering, University of the Basque Country (UPV/EHU), Plaza Europa 1, 20018 Donostia-San Sebastián, Spain; agnieszka.tercjaks@ehu.eus; 3 Centre of Polymer and Carbon Materials, Polish Academy of Sciences, 34 M. Curie-Sklodowska Street, 41-819 Zabrze, Poland; wsikorska@cmpw-pan.edu.pl (W.S.); bmendrek@cmpw-pan.edu.pl (B.M.); mkowalczuk@cmpw-pan.edu.pl (M.K.)

**Keywords:** branched polyurethanes, polyhydroxybutyrate, polylactide, chitosan, starch, degradability

## Abstract

One of the methods of making traditional polymers more environmentally friendly is to modify them with natural materials or their biodegradable, synthetic equivalents. It was assumed that blends with polylactide (PLA), polysaccharides: chitosan (Ch) and starch (St) of branched polyurethane (PUR) based on synthetic poly([R,S]-3-hydroxybutyrate) (R,S-PHB) would degrade faster in the processes of hydrolysis and oxidation than pure PUR. For the sake of simplicity in the publication, all three modifiers: commercial PLA, Ch created by chemical modification of chitin and St are called bioadditives. The samples were incubated in a hydrolytic and oxidizing environment for 36 weeks and 11 weeks, respectively. The degradation process was assessed by observation of the chemical structure as well as the change in the mass of the samples, their molecular weight, surface morphology and thermal properties. It was found that the PUR samples with the highest amount of R,S-PHB and the lowest amount of polycaprolactone triol (PCL_triol_) were degraded the most. Moreover, blending with St had the greatest impact on the susceptibility to degradation of PUR. However, the rate of weight loss of the samples was low, and after 36 weeks of incubation in the hydrolytic solution, it did not exceed 7% by weight. The weight loss of Ch and PLA blends was even smaller. However, a significant reduction in molecular weight, changes in morphology and changes in thermal properties indicated that the degradation of the samples should occur quickly after this time. Therefore, when using these polyurethanes and their blends, it should be taken into account that they should decompose slowly in their initial life. In summary, this process can be modified by changing the amount of R,S-PHB, the degree of cross-linking, and the type and amount of second blend component added (bioadditives).

## 1. Introduction

To obtain materials with suitable mechanical properties, which degrade after their service time, is a multi-faceted challenge. Polyurethanes (PURs) have played an essential role among high-strength polymers. Due to the ability of polyurethane chains to self-assemble and physically cross-link, these materials are characterized by exceptional mechanical resistance to tensile, bending, and friction [1,2]. Moreover, this structure brings high resistance to external factors, i.e., oils, water, temperature and microorganisms [3]. As a result, PUR waste can remain in the natural environment without degradation for many years. Therefore, the structure of PUR should be modified so that it retains its mechanical properties during its use, and at the end of its life cycle can be degraded in the environment. One way is to use biodegradable substrates to obtain a PUR material. Polyhydroxyalkanoate (PHA) and PLA are often called green polymers because they are both (bio)degradable and were obtained from renewable raw materials in a sustainable way [4,5]. Their synthetic counterparts are also highly susceptible to degradation.

PURs modified with biodegradable bioadditives are degradable, and additionally, are often biocompatible to the human body [6,7]. There are obtained PURs based on monosaccharides (such as fructose, glucose, mannitol, maltose, and sucrose) [8], polysaccharides [9,10,11], and their derivatives (such as mannitol [12]) or based on bioadditives, such as: vegetable oils [13], polyesters (such as PLA [14,15]), PHA (i.e., polyhydroxybutyrate [PHB]) [16], proteins [17] and others, such as lignin materials [18].

Biobased PURs are mainly used in medicine and agriculture [19,20,21,22]. However, if they degrade slowly, they could also be used in situations where their task is not to gradually release the active ingredient, e.g., in use as packaging material [23]. After the end of its service life (e.g., when frequent replacement of a given element is necessary due to its mechanical friction), the polyurethane waste could slowly degrade in the environment, reducing the amount of waste. Thus, the introduction of bioadditives to such PURs, which also have other applications, e.g., in packaging, should also be considered. Biomaterials and substrates should be selected so that PUR retains its functional properties for a long time and only degrades after the end of its life. In addition, the selection of substrates for PUR synthesis should be taken into account, so that when degraded in the environment, it is not only dispersed, but is actually biodegradable.

The degradability goes hand in hand with the structural characteristic of polymers, such as a low degree of crystallinity, high hydrophilicity and a low degree of cross-linking of polymers. The chemical structure of the polyurethane chains is also significant. For example, it is known that poly(esterurethane)s are susceptible to hydrolysis and the action of bacteria and fungi [19]. However, polycaprolactone is relatively hydrophobic, which makes it difficult to break down. This can be changed by partially replacing it with more hydrophilic chains, e.g., PHB [24] or poly(butylene succinate) diol (PBS diol) [25].

Apart from the structure of polymers, environmental conditions have a significant influence on the rate of their degradation. The most important degradation processes are hydrolysis and oxidation, followed closely by the activity of microorganisms [26].

The dependence of the physico-chemical properties on the chemical structure of the polyurethanes and their modification with biopolymers was discussed in previous papers [27,28]. The degree of cross-linking (related to the amount of PCL_triol_), the amount of R,S-PHB in the soft segments, and the presence of biopolymer in the blends had a significant influence on the crystallinity, morphology, hydrophilicity, density, as well as on thermal, sorption and mechanical properties of the investigated materials. As the degree of cross-linking, crystallinity, size and form of the crystallite, hydrophilicity, morphological structure, and even density affect the susceptibility of polymers to environmental factors, the same factors should also affect the degradability of the tested polyurethanes and their blends.

Since water is the factor that has the greatest influence on materials in the natural environment (also in the living organism), as described in this paper, a study of polyurethanes and their blends were treated with a phosphate solution with a neutral pH. Moreover, the action of oxidizing agents, including OH· radicals is important; therefore the samples were incubated in CoCl_2_/H_2_O_2_ solution [29,30].

This paper is a continuation of previous research [27,28] and discusses the degradability of branched polyurethanes with different contents of synthetic R,S-PHB and trifunctional PCL_triol_, and their blends with poly([D,L]-lactide) (PLA), St or Ch. These blends were prepared by direct blending of PUR solution with bioadditives. It is a simple and cheap method that does not require a complicated procedure. The influence of the hydrolytic and oxidizing environment on PURs and their blends was investigated. The dependence on the degree of degradation on the crystallinity of PUR, the amount of R,S-PHB and PCL_triol_ in the soft segments and the type of added modifier were analyzed.

## 2. Materials and Methods

Reagents necessary to obtain the R,S-PHB, PURs and their blends are summarized in Table 1.

### 2.1. Materials

Poly([R,S]-3-hydroxybutyrate) diol was synthetized via polymerization of β-butyrolactone, using anionic ring-opening polymerization, initiated by 3-hydroxybutyric acid sodium salt/18-crown-6 complex, at room temperature and terminated with 2-iodoethanol or 2-bromoethanol [31].

The polyurethanes were obtained by the two-step method according to the procedure described in our previous paper [18]. In brief, the prepolymer was synthesized from a polyol (PCL_diol_, PCL_triol_ and R,S-PHB) and from a diisocyanate (H_12_MDI) in the presence of OSn. In the second step, the chain extender (1,4-BD) was added to the DMF solution of the prepolymer. PURs were modified with biopolymers by physical blending of both components before forming the film. The exact methodology is discussed in a paper published earlier [28].

Table 2 shows the used nomenclature and the composition of the obtained polyurethanes and their blends.

Samples after hydrolysis are coded with the letter H, e.g., PUR 0/5 H, whereas after oxidation with the letter O, e.g., PUR 0/5 O.

### 2.2. Methods

For the degradability test, samples with an area of 1 ± 0.1 cm^2^ and a thickness of 0.1 ± 0.01 cm were cut. They were dried to a constant weight and weighed immediately before incubation in degradation solutions.

#### 2.2.1. Degradability in Hydrolytic Conditions

Sensitivity of PURs and their blends to hydrolytic conditions was estimated after their incubation in phosphate buffer solution (PBS, pH = 7.01) with sodium azide (as a bacteriostatic agent) for 4, 12, 24 and 36 weeks at 37 °C [32].

#### 2.2.2. Degradability in Oxidative Conditions

Degradation of polymer samples in oxidative conditions was carried out in 20% *w/w* hydrogen peroxide in 0.1 M cobalt chloride solution (high reactive solution) for 1, 2, 7 and 11 weeks at 37 °C [29,30]. Due to the extremely reactive oxidative acting of H_2_O_2_/CoCl_2_, the incubation time was shorter than for the hydrolysis.

After each incubation period, samples of polyurethanes and their composites were removed, rinsed with distilled water and dried to a constant weight (± 0.0001 g) at 37 °C in a vacuum drier. The values of experimental weight change were the arithmetic mean of 3–5 measurements. Degradability of polymers was estimated by observation of changes in sample mass, molecular weight, surface structure, and thermal properties after incubation of samples in the above-mentioned solutions and after drying them to a constant weight.

#### 2.2.3. Chemical Structure

Attenuated Total Reflectance Fourier Transform Infrared Spectroscopy (ATR FTIR) was used to determine the characteristic groups of polyurethanes. FTIR spectra were recorded with an attenuated total reflection (ATR Smart Orbit Accessory, Thermo Scientific, Madison, WI, USA) mode on a NICOLET 380 FTIR spectrometer (Thermo Scientific, Madison, WI, USA) with a diamond cell. A resolution of 4 cm^−1^ and the scanning range from 600 to 4000 cm^−1^ were applied, and 32 scans were taken for each measurement.

#### 2.2.4. Molecular Weight

The average molecular weight and molecular weight dispersity (M_w_/M_n_) number of the polymers were determined using gel permeation chromatography (GPC-MALLS) with a differential refractive index detector (Δn-2010 RI WGE Dr. Bures) and a multiangle laser light scattering detector (DAWN EOS from Wyatt Technologies). GPC was performed using the following set of columns: GRAM gel guard, GRAM 100 Å, GRAM 1000 Å and GRAM 3000 Å (Polymer Standard Service, Germany). The measurement was made in DMF supplemented with 5 mmol/L LiBr at 45 °C with a nominal flow rate of 1 mL/min. The sample solution was filtered prior to injection using an SRP 15 filter with a pore size of 0.20 µm (Sartorious, Germany). The polystyrene standards (Polymer Laboratories, UK), with narrow molecular mass distribution were used to generate a calibration curve. The results were evaluated using PSS WinGPC Unity software from Polymer Standard Service, Germany.

#### 2.2.5. Surface Topography

- Scanning electron microscopy (SEM): The sample surfaces before and after hydrolysis were analyzed by a Hitachi S-4800 scanning electron microscope (SEM) (Hitachi High-Technologies Corporation, Tokyo, Japan). Samples were metallically covered and scanned at an accelerated voltage of 10 kV at a working distance of 8 mm. Different zones of each sample were analyzed to ensure the morphology of each sample.
- Optical Microscopy(OM) micrographs were taken using a Nikon Eclipse E600W microscope (Mettler FP 82 HT, Melvile, NY, USA). The micrographs were collected with the software analySIS docu FIVE.

#### 2.2.6. Thermal Properties

Differential Scanning Calorimetry (DSC) measurements were performed using a Mettler Toledo DSC3+ (Mettler Toledo, Columbus, OH, USA). The investigated samples were first heated from −80 to 190 °C at 10 °C min^−1^, then cooled from 190 °C to −80 at 10 °C min^−1^, and finally, the melting was performed heating the samples up to 190 °C at 10 °C min^−1^. The experiments were conducted under a nitrogen flow of 10 mL/min using 5–10 mg samples in aluminum pans.

## 3. Results and Discussion

Polyurethane samples differing in the content of R,S-PHB and cross-linking, as well as their blend with polysaccharides (chitosan and starch) and PLA were incubated in a solution of phosphate buffer and oxidative solution CoCl_2_/H_2_O_2_.

### 3.1. Weight Changes

**Figure 1:**
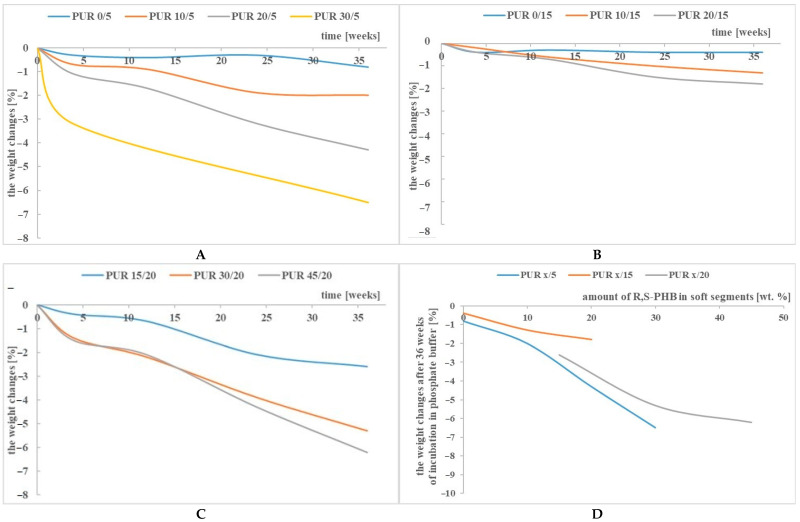
The weight changes of PUR x/5 (**A**), PUR x/15 (**B**) and PUR x/20 (**C**) after incubation in buffer solution, and dependence of the weight changes after 36 weeks of incubation on the amount of R,S-PHB in the structure of PURs soft segments (**D**).

Changes in the weight of PUR samples incubated in the hydrolytic solution are presented in Figure 1. The test was performed in a neutral solution; therefore, the hydrolysis reaction was not induced by a change in the pH of the environment. This degradation study was carried out on non-porous film specimens and in the absence of microorganisms, enzymes and any reactive compounds or radicals. The degradability of polymers in an inert environment is generally difficult. In our case, the potential acidic degradation products of polyurethane, which could accelerate its decomposition, were not left in the solution—with a noticeable decrease in pH, the buffer solution was replaced with a fresh one.

It is known that the scission of hydrolysable bonds and subsequent reactions lead to the formation of low molecular weight chains that are able to migrate from the polymer matrix to the degradation media, which results in mass loss [33]. However, the maximum weight loss of the samples after 36 weeks of incubation in the buffer did not exceed 6.5% (Figure 1A, Figure 1B and Figure 1C). However, the changes in the properties of the samples shown later in this article indicate that it could be related to the insolubility of the degradation products in water and does not reflect the actual degradation of the PCL based polyurethanes. Moreover, this slight loss in sample weight may only be apparent and may be due to the entrapment of water molecules in the polyurethane network.

Figure 1D shows the dependence of the susceptibility of samples to degradation dependent of the PUR structure after 36 weeks of incubation in a buffer solution. In any case, it was found that the hydrolysis susceptibility of PURs was closely related to the amount of R,S-PHB, as it increased with increasing R,S-PHB. The degree of cross-linking also has an influence on the degradation process and it was found that PUR samples with an amount of PCL_triol_ higher than 5% in the structure of the soft segments reduced the susceptibility to degradation (Figure 1D).

The dependence of the degradability of cross-linked polyurethanes with the soft segment made of PCL and PHB units on the amount of PHB has already been observed [24]. Hong et al. found that the presence of β-butyrolactone units in the soft segment of PUR decreased its crystallinity and resulted in the enhancement of its hydrolytic degradation [34].

PUR x/15 weight reduction was the lowest between all samples. Compared to PUR x/5 samples, it was obvious because PUR x/15 samples had a higher degree of cross-linking, and hence it was difficult to wash out degraded chains by “retaining” them with additional cross-links. However, increasing the degree of cross-linking in the case of PUR x/20 samples did not slow down their degradation after incubation in buffer solution compared to PUR x/15, but on the contrary, it accelerated. The elution of short polymer chains, formed after the degradation of the sample, is influenced by the “strength” of the interaction between them and the undegraded polymer network. These short chains are held in the network by chemical bonds (resulting from the use of a trifunctional polyol), but also by interactions such as van der Waals or hydrogen bonds. We suppose that, in the case of PUR x/15, the number of branching nodes and the resulting hydrogen bonds were so large that it made difficult to wash out the short chains, and thus the weight of the incubated samples did not decrease. However, a further increase in the amount of PCL_triol_ in PUR x/20 reduced the mobility of the chains, which could not self-assemble and create additional hydrogen bonds. Hence, the easier washing out of degraded chains. Thus, we supposed that the low sample weight reduction of PUR x/15 was a consequence of two effects of the overlap. It was a reason for the strange behaviour of PUR x/15.

**Figure 2:**
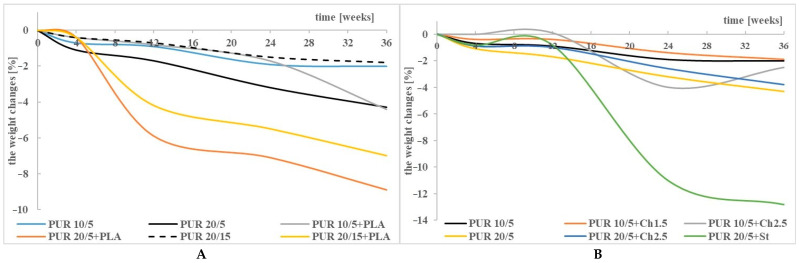
The weight changes of PURs and their blends with PLA (**A**), and Ch and St (**B**) after incubation in a buffer solution. For comparison, PUR 10/5, PUR 20/5 and PUR 20/15 are also shown.

The presence of Ch in the polyurethane network unexpectedly did not cause major changes in the weight of the incubated samples (Figure 2A). Firstly, this indicates an interaction between the PUR chains and Ch particles, and secondly, the ability of Ch to high water sorption could result in an apparent lack of weight loss, despite the fact that the samples were dried to dryness. In contrast, blending the PUR 20/5 with St significantly accelerated the kinetics of the degradation process (Figure 2B). The solubility of St in water and its swelling (visible in the first stage of sample incubation) loosen the structure of the PUR matrix, thus facilitating the penetration of water molecules and increasing the surface of PUR degradation.

**Figure 3:**
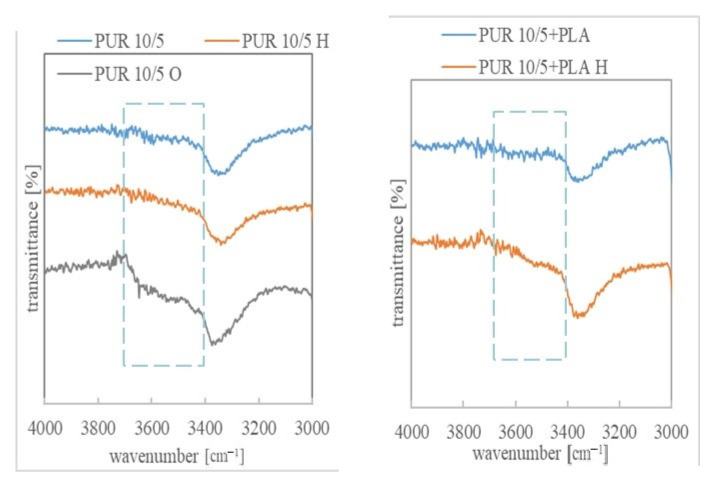
ATR-FTIR spectra in the range 3000-4000 cm^−1^ of PUR 10/5 and PUR 10/5+PLA, before and after degradation in hydrolytic (36 weeks) and oxidizing (7 weeks) solutions.

Blends of the PURs with PLA deteriorated faster than pure PUR 10/5, PUR 20/5 and PUR 20/15. The greatest changes in the mass of the samples were found for PUR 20/5+PLA, in which there was a higher amount of R,S-PHB than in PUR 10/5+PLA and a smaller amount of PCL_triol_ than in PUR 20/15+PLA. The results obtained by Heimowska et al. indicate that PLA is a polymer quite resistant to chemical hydrolysis [35], especially if the hydrolysis is carried out under neutral pH conditions [36,37]. The greatest difficulty in using this polymer as a biodegradable material for disposable products is that it degrades very quickly in industrial compost, but under natural conditions (in soil and water environment) its decomposition is very slow. However, the growing awareness of the public gives hope that this waste will be sent to a deliberate degradation site, and not to a random, wild dump. The obtained results indicate, however, that blending of PLA with low-branched polyurethanes accelerates their degradation, but due to the conditions prevailing during these studies, the kinetics of this process does not seem to be high. This is indicated by slight changes in the mass of the samples after incubation in the buffer solution. It should also be taken into account that water molecules could be trapped in the amorphous phase, and despite drying the samples after incubation to a constant mass, they remained in it, thus increasing the apparent mass of the samples. This could be the reason for these slight changes in sample weight (Figure 3A).

### 3.2. Molecular Weight

For the selected samples, the analysis of changes in their molecular weight after incubation in a hydrolytic solution was performed and summed in Table 3. The molecular weight of polyurethanes with a higher degree of cross-linking could not be determined due to their insolubility in the solvent used.

A decrease in the molecular weight of the polymer after incubation in a buffer solution is visible in the chromatogram as a line shift to lower molecular masses (Figure S3, blue line). This indicates that the PUR chains have been evenly cut into shorter chains, and together with that previously observed, no significant change in sample weight suggests that the cut chains are trapped in the polymer network. Hence, the shape of the chromatograms before and after incubation is very similar, without any visible changes in their broadening with similar dispersity (Table 3, Figure S3).

PUR 0/5 was characterized by the highest molecular weight among all the tested samples (Table 3). Its dispersity was lower after hydrolysis, but this could be related to the insoluble fraction that was filtered off in the case of the starting sample. In the rest of the samples, the dispersion of the molecular weight after hydrolysis was the same or higher. In the latter case the chains were cut and therefore the molar mass decreased and degraded to chains of varying length, and therefore M_w_/M_n_ increased.

After incubation in a buffer solution, the lowest molecular weight fractions were probably washed out from the PUR 0/5 samples, and the longest chains degraded, leading to a reduction in average molecular weight, with a decrease in dispersity (Figure S3).

A much higher reduction in molecular weight was observed for samples containing R,S-PHB in their structure (samples PUR 20/5 and PUR 30/5, Table 3). With an increase in the R,S-PHB content, the molecular weight of the polyurethane decreased. Furthermore, the reduction in their molecular weight after incubation was even greater compared to PUR 0/5. As discussed previously [27], the steric hindrance associated with the presence of a methyl group on the carbon next to the hydroxyl group in R,S-PHB makes this oligomer less reactive. This leads to a polyurethane with a lower molecular weight than PUR without R,S-PHB. Moreover, the presence of amorphous R,S-PHB increased the hydrophilicity [27] and decreased the crystallinity (Table 4) of the PURs, which made it easier for water to penetrate into the sample mass and hydrolyze the macrochains to shorter ones. As a result, a significant reduction in molecular weight was found for both PUR 20/5 and PUR 30/5 after 36 weeks of incubation (Table 3, Figure S3).

The highest changes in molecular weight were found for the PUR 20/5+PLA blend. While the samples did not change their weight to a large extent (Figure 2A), for the blends of PUR with PLA, the higher M_w_ decreasing compared to pure PUR was observed (Table 3, Figure S3).

### 3.3. Chemical Structure

The degradation progress was also studied by assessing the change in their chemical structure after 36 weeks of incubation in a buffer solution and after 11 weeks of oxidizing.

The ATR-FTIR spectra of PURs and their blends before degradation contained of typical bands associated with urethane groups present at: 3300 cm^−1^−3380 cm^−1^ (stretching vibration band of N-H), about 1721 cm^−1^ correlated to the overlapped carbonyl group of esters and urethane linkages, about 1521 cm^−1^ (urethane N-H bending + C-N), about 1239 cm^−1^ (C-N stretching), and about 1045 cm^−1^ (urethane C-O-C stretching) and C-O-C band of PCL/R,S-PHB units. According to Bil et al., the presence of these bonds in the spectra after degradation in buffer solution indicated good stability of the urethane linkages in hydrolytic environment [33]. The 1362 cm^−1^ peak was associated with CH_2_ twisting, wagging, and scissoring vibrations in polyols, whereas the bands in the range of 2970–2800 cm^−1^ arose from C-H stretching vibration in methyl, methine, and methylene [20,38].

Changes in the chemical structure of polyurethanes under the influence of external factors can be observed, especially by comparing the bands corresponding to the stretching vibrations of the -NH and -C=O groups in PURs, before and after incubation in degradation solutions. The individual lengths of these strands for the PURs and their blends are shown in Table S1. Shifts of these bands towards higher wavelength values indicate the dissociation of hydrogen bonds due to degradation factors. However, an increase in the number of hydrogen bonds (visible by shifting the bands towards lower values) may also indicate a degradation progress due to structural changes formed during samples incubation in buffer and oxidizing solutions. While changes in the band corresponding to the tensile vibrations of the carbonyl groups are minimal, the wave number of the -NH group band of urethane changed significantly after the samples were incubated in the tested solutions (Table S1).

**Figure 4:**
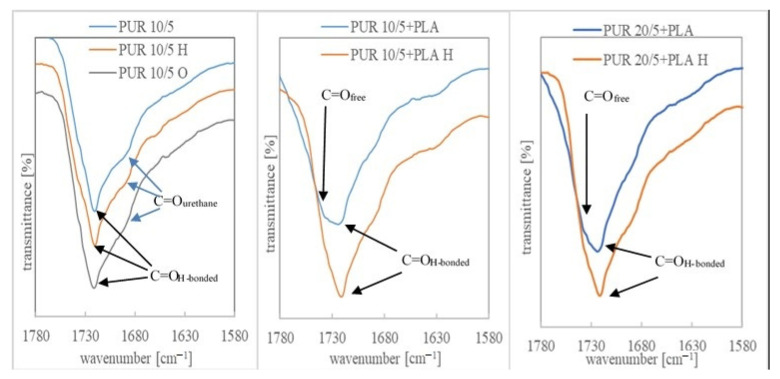
ATR-FTIR spectra in the range 1580-1780 cm^−1^ of PUR 10/5, PUR 10/5+PLA and PUR 20/5+PLA, before and after degradation in hydrolytic (36 weeks) and oxidizing (7 weeks) solutions.

**Figure 5:**
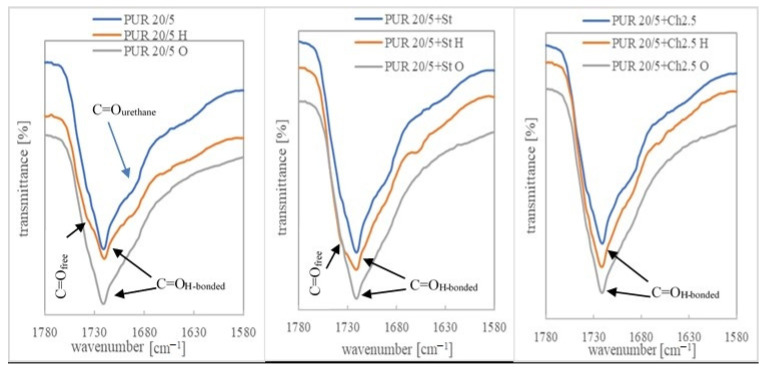
ATR-FTIR spectra in the range 1580-1780 cm^−1^ of PUR 20/5, PUR 20/5+St and PUR 20/5+Ch2.5, before and after degradation in hydrolytic (36 weeks) and oxidizing (1 week) solutions.

Moreover, the spectra of the incubated samples showed a characteristic broad peak in the region of 3400 cm^−1^-3700 cm^−1^. It indicates the formation of -OH groups as a result of ester and urethane group degradation [39]. This band was more intense for the samples after incubation in the oxidizing solution, which corresponds to a much higher weight loss in this aggressive solution than in the buffer. The ATR-FTIR spectra in the range 3000 cm^−1^-4000 cm^−1^ and 1580 cm^−1^-1780 cm^−1^ are shown for the example of PUR 10/5, PUR 20/5 and their blends with PLA, Ch and St, before and after degradation in a buffer and oxidizing solution (Figure 3, Figure 4 and Figure 5). An exemplary spectrum over the full wavelength range is provided in the Supplementary Material Section (Figure S1).

Bands indicating the presence of non-hydrogen bonded ester carbonyl groups (from polyols) were overlapped with larger bands corresponding to the hydrogen bonded C=O groups. Only in the case of PLA blends, the number of free carbonyl groups was much greater, which could be seen in the ATR-FTIR spectra (Figure 4 and Figure S2). This indicated, firstly, a disturbance of the interaction between ester and urethane moieties after the addition of PLA, and secondly, that the new ester groups introduced with the PLA chains were not compatible with urethane and no hydrogen interactions were formed between them. Interestingly, after incubation in a hydrolytic solution, the bands corresponding to the C=O_H-bonded_ groups were much more pronounced than the unbound groups, which indicated that the presence of water molecules affected the compatibility of PLA with PUR.

In other samples, it was found that after incubation in a buffer solution some of the hydrogen bonds between the ester and urethane groups had dissociated and the bands corresponding to the C=O_free_ groups became more clearly visible (Figure 5). The presence in this region of the spectrum of bands corresponding to the vibrations of free C=O groups and bounded by hydrogen bonds was indicated by other authors [40,41].

### 3.4. Thermal Properties

During the degradation of polymers, many processes occur in their structure: long chains are cut into shorter ones, which can cause secondary cross-linking of the polymer; the mobility of the chains increases, which can lead to an increase in crystallinity and an increase in the glass transition temperature; and finally, the short chains are washed out of the polymer mass. In the latter case the polymers structure is loose and subsequent absorption of water molecules occurs. This, along with degradation factors, accelerates the degradation process. The amorphous phase of the polymer is more susceptible to degradation [34], therefore an apparent increase in the crystalline phase can be observed in the DSC thermograms, which is indicated by an increase in the enthalpy of melting.

Microstructural changes in PURs and their blends after incubation in a buffer solution were observed by DSC. Thermograms confirmed the semicrystalline nature of PURs, where the crystalline phase was formed in the PCL fraction. The glass transition temperature of the soft segments’ amorphous phase (T_g_), as well as their melting temperature (T_m_) were visible on the DSC thermograms (Table 4, Table 5 and Table 6). The melting enthalpy (ΔH) was also determined.

The lowest values of glass transition temperature (T_g_) were noted for PURs without R,S-PHB. These values increased after introducing R,S-PHB into the PUR structure (Table 4), because of relatively high T_g_ of R,S-PHB (−5.5 °C). The presence of one T_g_ on DSC thermograms indicated on miscibility of these two oligomerols and increasing the R,S-PHB quantity increased the glass transition temperature [27].

The lowest changes in thermal properties after degradation were found for the PUR 0/15 sample. This is in agreement with the results of the weight loss of the samples (Figure 1 D). The lack of PUR modification by R,S-PHB and biopolymers as well as a relatively high degree of cross-linking were the reasons for the highest resistance of this material to the hydrolytic environment.

The generally increasing of the soft segments T_g_ indicated the stiffening of the chains at the end of degradation. This stiffening may result from the involvement of chains in the formation of crystalline forms as well as from secondary cross-linking [42]. Initially, under the influence of water molecules penetrating between the chains their mobility increased. As a result, the chains could reorder and form new crystal forms. In addition, the short chains formed after polymer hydrolysis were more mobile, which could also lead to their separation and the formation of new crystalline formations. Both factors could cause the formation of crystallites with different melting points, as can be seen in the DSC thermograms, such as in the case of PUR 30/5 H (Figure S6). The total enthalpy of this crystalline phase was slightly greater after 36 weeks of degradation than before.

Generally, it can be said that the crystallinity only decreased after incubation in a hydrolytic solution for PURs without R,S-PHB. In all other cases, the enthalpy of melting increased, sometimes to a great extent. Similar observations of the increase in crystallinity of PUR based on poly(caprolactone/lactide-*co*-glycolide) after degradation were reported by Bil and co-workers [33]. Mondal and Martin also noted the increase of crystallinity of the PCL-based PUR after hydrolysis in phosphate buffer solution [38]. They stated that the reason was an easy diffusing of the water molecules into the amorphous region of the polymer, thus as a consequence the hydrolytic degradation occurred preferably in the amorphous region rather than the crystalline region.

Interestingly, blending the low cross-linked PURs with a small amount of polysaccharides lowered the T_g_ (Table 5). This suggests that the microphase separation between soft and hard polyurethane domains was improved [42]. Incubation of the samples in the hydrolytic solution shifted the T_g_ towards higher values, and what is more, in some blends, such as for PUR 20/5+St, no glass transition was found on the DSC thermogram (Table 5, Figure S5). This transition is not visible because it probably has been superimposed by a large endopeak (115.5 J/g) related to the melting of the soft segment crystalline phase. Under the influence of water, the structure of most PURs and some of their blends developed crystalline forms different than before incubation. The differences in the size of the crystallites and the type of their arrangement caused them to melt at different temperatures. Such a significant increase in PUR 20/5+St crystallinity after degradation in the buffer was surprising. Starch could undergo crystallinity due to reorganization connected to the action of water, but its crystalline phase had a melting point of about 85 °C. Besides, only a small amount of St (2.5 wt.%) was used in the blend, so the observed high enthalpy of melting could not be a result of the melting of starch crystallites. Presumably, the starch particles acted as nucleating agents of PUR soft segments.

Chitosan particles had a similar nucleating effect on the chains of the soft segments. However, increasing the amount of amorphous R,S-PHB decreased the crystallite formation tendency of the PCL chains in PUR 20/5+Ch2.5 and their melting enthalpy only increased slightly after incubating the samples in the buffer.

On the DSC thermograms of the second heating cycle of PUR/PLA blends, the presence of the second T_g_ was observed at a temperature of about 46 °C (Figure S4). It indicated incompatibility between PLA and the soft segments of PUR. As can be seen, T_m_ read from the first heat is superimposed on the T_g_ of PLA seen in the second heat. Therefore, since we performed the first heating cycle during the DSC test for the sample after the hydrolysis, the T_g_ for PLA cannot be seen. The first heating cycle is chosen to see what happened to the sample during incubation in the degradative solution.

It was observed that T_g_ of soft segments increased significantly after the samples incubation in the hydrolytic solution. Only in the case of PUR 10/5+PLA T_g_ was not visible, possible due to the superposition with a large melting peak of the soft segments. Moreover, the melting peaks were split into two for some samples, with a lower and higher temperature than before incubation (Table 6).

As mentioned earlier, the increase in the crystallinity of the samples, observed as an increase in the enthalpy of melting in DSC thermograms, after incubation in the hydrolytic solution, may result from the degradation of the amorphous phase. However, it may also be a consequence of the increased mobility of the chains under the influence of the plasticizing effect of water molecules, which leads to their arrangement and formation into crystalline forms. This increase in the enthalpy of melting was particularly evident in PUR blends with PLA (Table 6). However, these changes were not as spectacular as in the case of polysaccharides (Table 5).

Despite these high changes in the thermal characteristics of PURs and their blends, the relatively low weight loss of the samples (Figure 1 and Figure 2) could be related to the insolubility of the degradation products in water and did not reflect the actual degradation of the PCL based PURs.

### 3.5. Microscopic Observation

**Figure 6:**
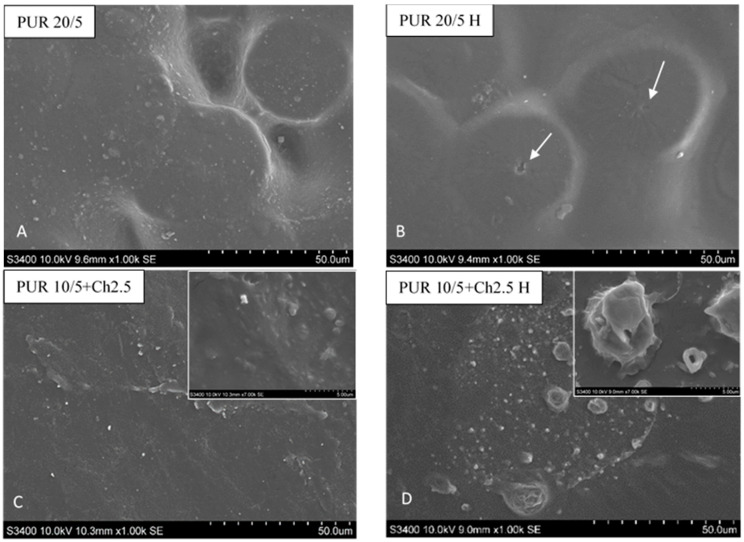

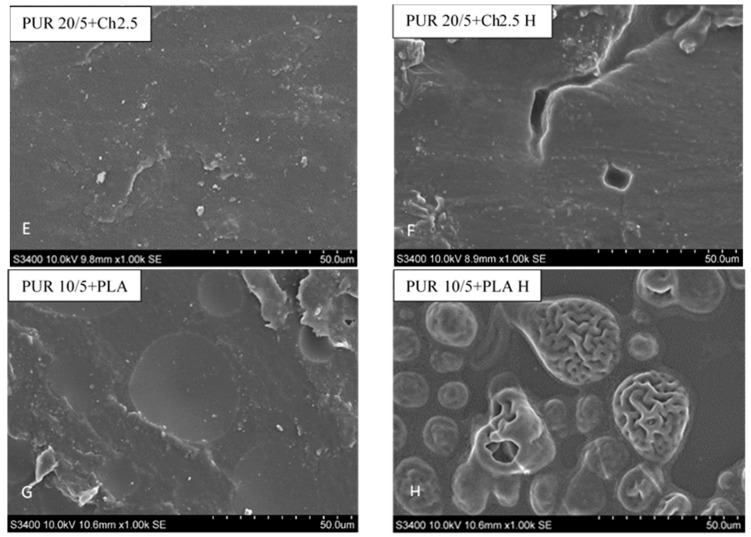
SEM images of the surface of PUR 20/5 (**A**,**B**), PUR 10/5+Ch2.5% (**C**,**D**), PUR 20/5+Ch2.5% (**E**,**F**) and PUR 10/5+PLA (**G**,**H**) before and after incubation in the buffer solution. The samples with Ch were analyzed after 36 weeks of incubation, whereas PUR 10/5+PLA was after 12 weeks.

**Figure 7:**
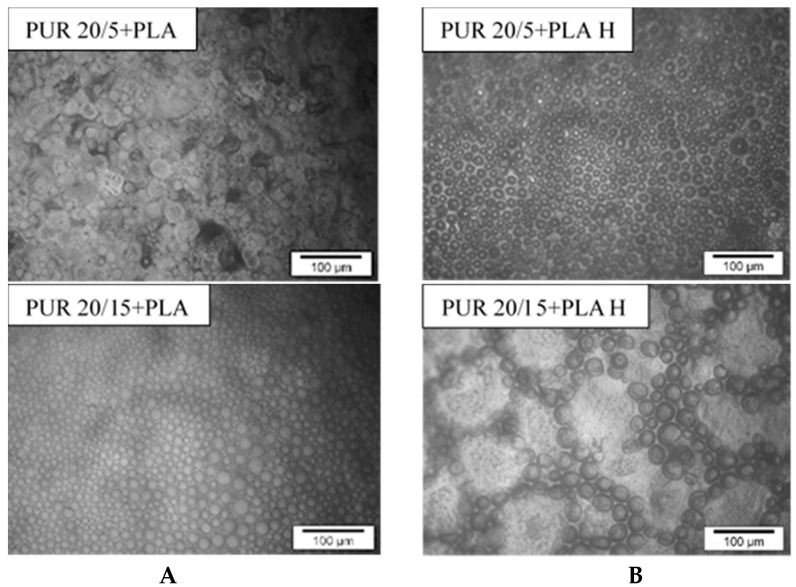
Microscopic images of the surface of PUR 20/5+PLA and PUR 20/15+PLA samples before (**A**) and after 12 weeks (**B**) of incubation in phosphate buffer.

**Figure 8:**
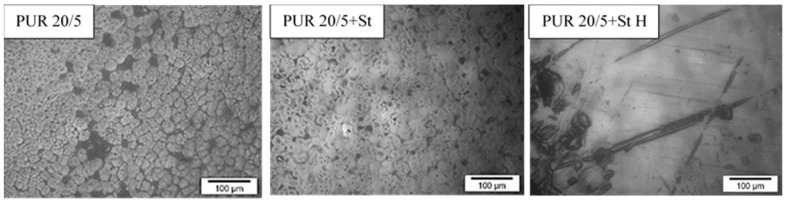
Optical microscope micrographs of the surface of PUR 20/5, PUR 20/5+St and PUR 20/5+St H.

The degradation process was also investigated by the samples surface observation under SEM (Figure 6) and optical microscope (Figure 7 and Figure 8).

Even if there are changes in the structure, thermal properties and mass of PUR 20/5 after incubation were not large, and the SEM images show cracks on the sample surface (marked with white arrows) (Figure 6).

The SEM images of PUR 10/5+Ch before hydrolysis showed that the chitosan particles were tightly bound to the PUR matrix and were surrounded by PUR chains. There were no visible spaces between the matrix and Ch particles. This indicated a close relationship between these fractions. These clear color contrasts in the appearance of the matrix and chitosan microparticles, and their tight connection, were also noted by Bil and co-workers in Reference [33]. However, after 36 weeks of incubation, individual Ch particles were extracted, separated from the matrix (in higher magnification inside it is clear visible); the interaction between Ch and PUR chains was clearly weakened. The water diffusion into PUR/Ch blend caused a deterioration bonding between both components. The chitosan particles appeared swollen with water. The similar observation was done for PUR 20/5+Ch. Moreover, there were observed cracks and defects on the sample surface. It was presumed that the chitosan particles were washed out during the incubation. However, these cracks could also indicate the hydrolysis of the esters in the buffer solution [43].

In line with our earlier observations, due to the incompatibility of PLA and PUR chains, polylactide drops are dispersed in the polyurethane matrix [44]. This morphology is called the sea-island structure [45]. The incompatibility shown on SEM images is in line with the DSC observations (Figure S4).

The incubation of samples with PLA in a hydrolytic solution completely changed the structure of the blend (Figure 6G and Figure 6H). The structure of the PLA droplets was homogeneous and smooth before degradation in phosphate buffer. However, after 36 weeks of incubation, the amorphous PLA swelled, what was resulted in intense folding in the entire mass of the polymer. These droplets took on a three-dimensional character. The hydrolytic degradation occurs in the bulk of polymers, causing chain scissions and molecular weight reducing [46]. After polymers swelling it was possible for water molecules to penetrate deeply into the polymer bulk. This caused a slow leaching of hydrolysed polymer chains, and consequently the weight loss of PUR blends with PLA (Figure 2A). The loosening of the PLA chains and the penetration of water into the droplets, embedded in the PUR network, simultaneously increased the surface of the interaction of water with the PUR chains. It is therefore a likely mechanism for increasing the degradability of PUR blends with degradable natural additives.

Interesting changes in the arrangement of round PLA inclusions were observed on the surface of the PUR 20/15+PLA sample after buffer incubation (Figure 7). Under the influence of solution water, these inclusions slightly increased in diameter and formed into characteristic circles. This indicates that both polymers have become even more incompatible.

There were no differences in PUR 20/5 and PUR 20/5+St morphology (Figure 8). They were both patterned and rough. However, significant changes were visible on the surface of the PUR 20/5+St samples incubated for 36 weeks in buffer solution. Clear black depressions/holes were visible, presumably indicating degradation of the starch chains. These significant changes in morphology confirmed both the changes in thermal properties occurred under the influence of water and the weight loss of the samples. Although the weight loss of the PUR 20/5+St samples was not significant after 36 weeks of incubation in a solution of hydrolysis, the changes of the surface, as well as a significant shift of melting temperature clearly indicated the progressive degradation of the material.

### 3.6. Degradation in Oxidative Solution

The susceptibility of samples to the action of free radicals in an oxidizing environment was also checked (Table 7).

As was expected, the weight loss of PUR samples and their blends under the influence of oxidizing agents were much higher than after incubation in the hydrolytic solution. The used solution was much more aggressive than the neutral buffer solution. According to Feng and Li, hydrogen peroxide and cobalt(II) ions undergo the following Habere Weiss reaction and produce reactive hydroxyl radicals HO· [30]. These radicals induce a series of oxidative reactions in the PUR chains, and consequently chain scission and/or crosslinking. Due to Christenson et al. 24 days treatment of PUR samples in this solution closely reproduced all the spectral changes observed after 12 months implantation in the living body [29].

Therefore, at the end of the experiment, most of the incubated samples disintegrated. Even though the some samples did not disperse after the last test time, they were flaky and sticky, indicating a large change in their molecular weight and gradual dissolution of the short chain degradation products.

The degradability was strictly dependent on three factors: the amount of R,S-PHB and PCL_triol_ in the soft segments, and the type of biopolymer additive. The susceptibility of the samples to degradation clearly increased after an increase in the amount of R,S-PHB in the chains of soft segments. Whereas samples degradability decreased after an increase in the degree of cross-linking, connected with the increasing of PCL_triol_ amount. Most of the blend samples disintegrated in the second week of incubation. The highest weight loss was found in the first week of the experiment for the PUR 20/5+St system. Meanwhile, the slowest degradation was in the case of PUR 10/15+PLA—a sample with a small amount of R,S-PHB, a higher degree of cross-linking and the addition of the probably slowest degrading under these conditions of biopolymer (PLA).

**Figure 9:**
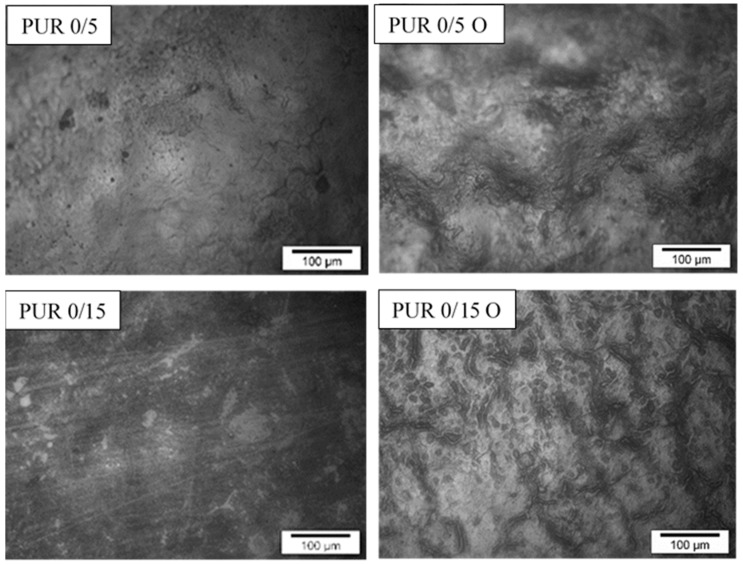
Microscopic micrographs of PUR 0/5 samples before and after 11 weeks of incubation in the oxidizing solution.

As most of the samples were sticky after one week of incubation, microscopic micrographs could be made only for a few samples of the tested polymers. These were mainly samples without R,S-PHB, or with a small amount of it, or with a high degree of cross-linking. While the weight loss of samples not containing R,S-PHB (PUR 0/5 and PUR 0/15) in their structure was negligible (Table 5), these samples were deformed after incubation in the oxidizing solution. Microscopic images show changes in the morphology of their surface (Figure 9). The surface of the samples very often became wavy (such as in the case of PUR 0/5) and heterogeneous (e.g., PUR 0/15). The increased heterogeneity of the surface and its ripple probably favor the acceleration of the degradation process by increasing the surface of the interaction. In some cases, the abrupt, rapid weight loss appeared, such as for PUR 20/5, when after 1 week of incubation only 1% weight loss was found, and after the second week, the samples completely disintegrated.

## 4. Conclusions

The aim of the study was to assess the dependence between the presence of R,S-PHB and PCL_triol_ in the structure of branched PURs, and the degradability under hydrolytic and oxidative conditions. The influence of PURs modification with biodegradable additives—Ch, St and PLA, on the sensitivity of these materials to degradation environments was also determined.

While the weight losses of the samples incubated in the hydrolytic solution were not high, the significant reduction in molecular weight, changes in the surface morphology and in thermal properties indicated a high process of degradation. The relatively low weight loss of the samples (no more than 6.5 wt.%) could be related to the insolubility of the degradation products in water and did not reflect the actual degradation of the PCL based polyurethanes. Moreover, this slight loss in sample weight may only be apparent and may be due to the entrapment of water molecules in the polyurethane network.

This study demonstrated that increasing the amount of R,S-PHB and decreasing the PCL_triol_ accelerated the degradation process. Moreover, blending of PUR with Ch, St and PLA increased the susceptibility of the tested materials to degradation in both hydrolytic and oxidizing environments. The degradation of the samples in the strong oxidizing solution was much faster than in the neutral phosphate buffer solution. After 2 weeks of incubation in oxidative solution, most of the blend samples were disintegrated.

It is assumed that, by handling the amount of R,S-PHB and PCL_triol_, and the type and amount of biopolymers, the polyurethane materials with appropriate mechanical properties and the desired/designed degradation rate can be obtained.

## Figures and Tables

**Table 1:** List of chemicals.

Chemical	Producer	Chemical	Producer
ß-butyrolactone	Aldrich, St. Luis, MO, USA	Tin(II) octanoate (OSn)	Alfa Aestar, Karlsruhe, Germany
ß-18-crown-6 complex	Aldrich, St. Luis, MO, USA	Poly([D,L]-lactide) (PLA), M_n_ 18,000–28,000	Aldrich, Steinheim, Germany
2-iodoethanol and 2-bromoethanol	Aldrich, St. Luis, MO, USA	Pea starch (St)	HengshuiFuqiao Starch Co., Hengshui, China
Poly(ε-caprolactone)diol (PCL_diol_),M_n_ 1900	Aldrich, St. Luis, MO, USA	Chitosan (Ch), Mn 171,000, degree of deacetylation 97%	MRI Gdynia, Gdynia, Poland
Poly(ε-caprolactone)triol (PCL_triol_), M_n_ 900	Aldrich, St. Luis, MO, USA	Phosphate buffer (pH 7.01)	Chempur, Piekary Śląskie, Poland
4,4′-methylene dicyclohexyl diisocyanate (H_12_MDI)	Aldrich, St. Luis, MO, USA	H_2_O_2_ (30%)	Chempur, Piekary Śląskie, Poland
1,4-butanediol (1,4-BD)	Aldrich, Steinheim, Germany	CoCl_2_	Chempur, Piekary Śląskie, Poland
N,N’-dimethylformamide (DMF)	POCh, Gliwice, Poland		

**Table 2:** Composition of polyurethanes and their blends.

Sample	Percentage of Oligomerol in Soft Segments [wt.%]			Percentage of Biopolymer in Blend [wt.%]		
	R,S-PHB	PCL_triol_	PCL_diol_	Ch	PLA	St
PUR 0/5	0	5	95	0	0	0
PUR 10/5	10	5	85	0	0	0
PUR 20/5	20	5	75	0	0	0
PUR 30/5	30	5	65	0	0	0
PUR 0/15	0	15	85	0	0	0
PUR 10/15	10	15	75	0	0	0
PUR 20/15	20	15	65	0	0	0
PUR 15/20	15	20	65	0	0	0
PUR 30/20	30	20	50	0	0	0
PUR 45/20	45	20	35	0	0	0
PUR 10/5+Ch1.5	10	5	85	1.5	0	0
PUR 10/5+Ch2.5	10	5	85	2.5	0	0
PUR 10/5+PLA	10	5	85	0	5	0
PUR 20/5+Ch2.5	20	5	75	2.5	0	0
PUR 20/5+PLA	20	5	75	0	5	0
PUR 20/5+St	20	5	75	0	0	2.5
PUR 20/15+PLA	20	15	65	0	5	0

**Table 3:** Molecular weight of PUR 0/5, PUR 20/5, PUR 30/5 and PUR 20/5+PLA samples before and after 36 weeks of degradation in buffer solution.

Sample	M_n_[Da]	M_w_/M_n_	M_n_ Reduction After Hydrolysis [%]
PUR 0/5 *	62 000	4.1	-
PUR 0/5 H *	49 000	3.4	21.0
PUR 20/5	27 000	2.2	-
PUR 20/5 H	9 700	2.6	64.1
PUR 30/5	17 500	2.2	-
PUR 30/5 H	5 000	2.2	71.4
PUR 20/5+PLA	26 000	2.4	-
PUR 20/5+PLA H	5 900	2.5	77.3

* sample PUR 0/5 was dissolved at 70 °C for 5 days. In both the PUR 0/5 and PUR 0/5H samples, when dissolved in DMF, there was an undissolved fraction. Before the measurement, the samples were filtered through a 0.2 µm filter and thus the insoluble fraction was removed.

**Table 4:** Thermal properties of PURs before and after incubation in the buffer solution.

Sample	T_g_ [°C]	T_m_ [°C]	ΔH [J/g]
PUR 0/5	−43.1	47.8	20.9
PUR 0/5 H	−41.6	44.5/49.6	16.3
PUR 10/5	−24.5	47.5	27.4
PUR 10/5 H	−16.0	44.0/56.0	52.6
PUR 20/5	−18.0	50.4	25.8
PUR 20/5 H	−14.2	54.9	32.5
PUR 30/5	−19.7	47.7	12.8
PUR 30/5 H	−14.0	44.4/55.2	15.8
PUR 0/15	−45.3	46.2	0.9
PUR 0/15 H	−42.1	42.9	0.3
PUR 10/15	−36.6	47.3/57.3	5.5
PUR 10/15 H	−24.0	42.3/55.7	17.7
PUR 20/15	−33.0	51.1	0.8
PUR 20/15 H	−14.0	47.0/57.0	23.8
PUR 15/20	−39.4	47.1	7.7
PUR 15/20 H	−22.4	45.0/56.9	12.6
PUR 30/20	−11.5	47.5	10.1
PUR 30/20 H	−9.5	44.7/56.0	10.0
PUR 45/20	−8.9	47.7	3.6
PUR 45/20 H	−2.4	44.2/57.2	6.9

**Table 5:** Thermal properties of PURs and their blends with polysaccharides before and after incubation in the buffer solution.

Sample	T_g_ [°C]	T_m_ [°C]	ΔH [J/g]
PUR 10/5	−24.5	47.5	27.4
PUR 10/5 H	−16.0	44.0/56.0	52.6
PUR 10/5+Ch1.5	−32.5	50.8	17.3
PUR 10/5+Ch1.5 H	-	2.4	66.6
PUR 10/5+Ch2.5	−36.7	52.5	13.1
PUR 10/5+Ch2.5 H	-	7.1	79.4
PUR 20/5	−18.0	50.4	25.8
PUR 20/5 H	−14.2	54.9	32.5
PUR 20/5+Ch2.5	−24.9	51.8	13.1
PUR 20/5+Ch2.5 H	−24.1	45.0	18.7
PUR 20/5+St	−26.7	51.1	11.9
PUR 20/5+St H	-	3.3	115.5

**Table 6:** Thermal properties of PURs and their blends with PLA before and after incubation in the buffer solution.

Sample	T_g_ [°C]	T_m_ [°C]	ΔH [J/g]
PUR 10/5	−24.5	47.5	27.4
PUR 10/5 H	−16.0	44.0/56.0	52.6
PUR 10/5+PLA	−35.3	48.9/79.4	19.9
PUR 10/5+PLA H	-	7.3	79.2
PUR 20/5	−18.0	50.4	25.8
PUR 20/5 H	−14.2	54.9	32.5
PUR 20/5+PLA	−29.5	50.1	10.1
PUR 20/5+PLA H	−15.0	45.0/54.0	27.1
PUR 20/15	−33.0	51.1	0.8
PUR 20/15 H	−14.0	47.0/57.0	23.8
PUR 20/15+PLA	−32.6	49.7	0.2
PUR 20/15+PLA H	−15.0	47.0/52.0	19.3

**Table 7:** The weight changes of samples after their incubation in oxidative solution.

Sample	The Weight Changes [%] with Time of Incubation [Weeks]			
	1	2	7	11
PUR 0/5	−0.2	−0.1	−0.4	−0.8
PUR 10/5	−1.3	0.1	−1.9	disintegration
PUR 20/5	−1	disintegration		
PUR 30/5	−2.1	disintegration		
PUR 0/15	0.0	−0.1	−0.5	−1.2
PUR 10/15	0.1	−0.1	−1	−38.1
PUR 20/15	0.1	0.0	−1.3	disintegration
PUR 15/20	−1.1	−1.6	−2.1	−40
PUR 30/20	−1.7	−1.7	−5.6	disintegration
PUR 45/20	−2	−2	−13	disintegration
PUR 10/5+Ch1.5	−2	disintegration		
PUR 10/5+Ch2.5	−5.4	disintegration		
PUR 10/5+PLA	0.2	−0.1	−12.7	disintegration
PUR 20/5+Ch2.5	−29.3	disintegration		
PUR 20/5+PLA	−6.1	disintegration		
PUR 20/5+St	−6.3	disintegration		
PUR 20/15+PLA	−0.7	−1.1	−5	disintegration

## Data Availability

No data other than that shown in the manuscript and in Supplementary Materials has been reported.

## Supplementary Materials

The following are available online at https://www.mdpi.com/article/10.3390/polym13081202/s1, Figure S1: ATR-FTIR spectra of PUR 10/5 before and after incubation in hydrolytic and oxidative solutions, Figure S2: ATR-FTIR spectra in the range 1580-1780 cm^−1^ of PUR 20/15 and its blend PUR 20/15+PLA, before and after degradation in hydrolytic (36 weeks) and oxidizing (7 weeks) solutions., Figure S3: Chromatograms (RI traces) of PUR 0/5, PUR 20/5, PUR 30/5 and PUR 20/5+PLA before (red line) and after (blue line) 36 weeks of incubation in hydrolytic solution (DMF, 1 mL/min), Figure S4: DSC thermogram of PUR 10/5+PLA heated from −80 °C to 180 °C (1 heating), cooled to −80 °C and again heated to 180 °C (2 heating), Figure S5: DSC thermogram of PUR 20/5+St before and after incubation in buffer solution, Figure S6: DSC thermogram of PUR 20/5 and PUR 30/5 before and after incubation in buffer solution, Table S1: Wavenumbers of the stretching vibrations of the -NH and -C=O groups of PURs and their blends before and after incubation in buffer and oxidizing solutions.
