# Supplementary material for: Degradability of Polyurethanes and Their Blends with Polylactide, Chitosan and Starch
Source: Polymers (Basel). 2021 Apr 8;13(8):1202. doi: 10.3390/polym13081202 (PMC8068122; doi:10.3390/polym13081202)
Supplement: Supplementary file 1 [file polymers-13-01202-s001.pdf]

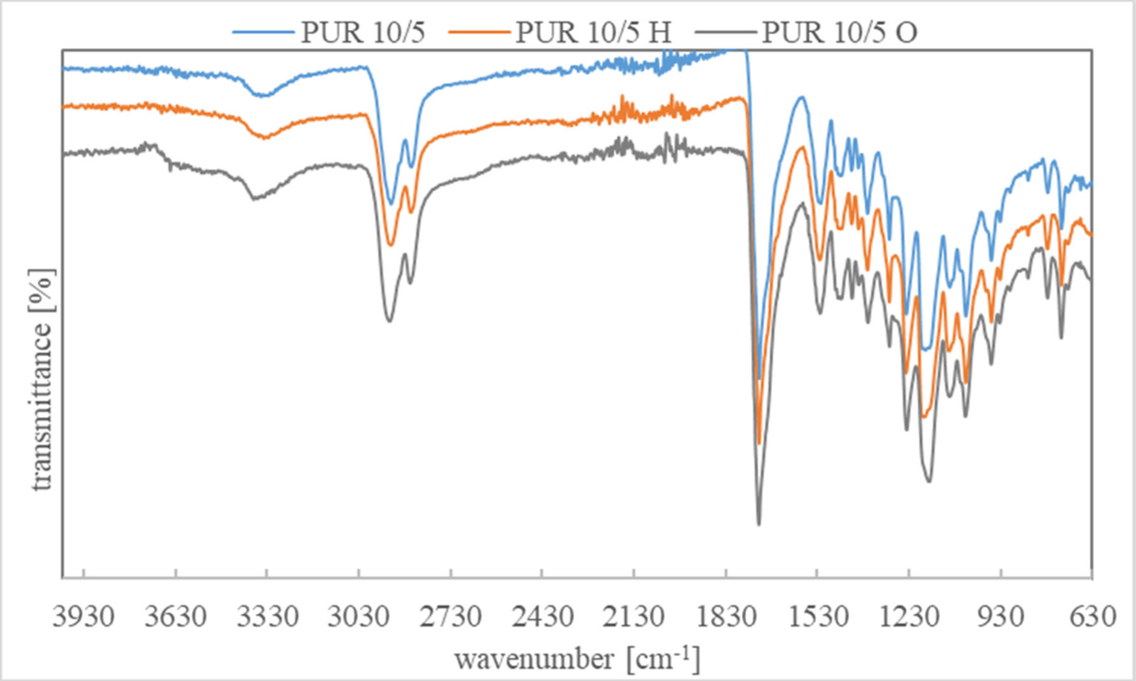

Figure S1. ATR-FTIR spectra of PUR 10/5 before and after incubation in hydrolytic and oxidative solutions

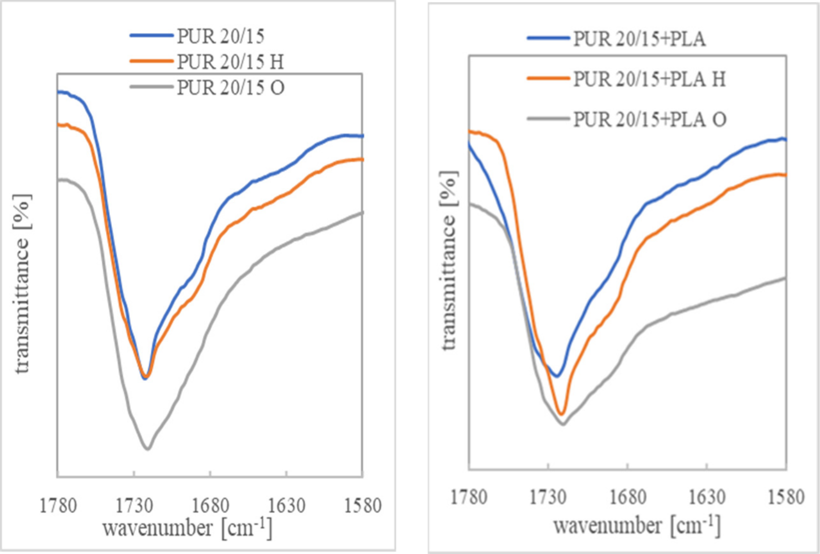

Figure S2. ATR-FTIR spectra in the range 1580-1780 cm^{-1} of PUR 20/15 and its blend PUR 20/15+PLA, before and after degradation in hydrolytic (36 weeks) and oxidizing (7 weeks) solutions.

Table S1. Wavenumbers of the stretching vibrations of the -NH and -C=O groups of PURs and their blends before and after incubation in buffer and oxidizing solutions

Sample	stretching vibration -NH group [cm^{-1}]	stretching vibration -C=O group [cm^{-1}]	Sample	stretching vibration -NH group [cm^{-1}]	stretching vibration -C=O group [cm^{-1}]
PUR 0/5	3366.9	1721.7	PUR 10/5+Ch1.5%	3348.3	1721.1
PUR 0/5 H	3340.6	1721.6	PUR 10/5+Ch1.5% H	3359.6	1721.2
PUR 0/5 O	3345.6	1720.9	PUR 10/5+Ch1.5% O	3354.2	1721.0
PUR 10/5	3351.4	1720.7	PUR 10/5+Ch2.5%	3363.3	1721.7
PUR 10/5 H	3341.4	1720.4	PUR 10/5+Ch2.5% H	3350.4	1720.9
PUR 10/5 O	3366.6	1721.0	PUR 10/5+Ch2.5% O	3342.8	1721.2
PUR 20/5	3363.0	1720.9	PUR 10/5+PLA	3357.9	1724.2
PUR 20/5 H	3341.7	1720.2	PUR 10/5+PLA H	3360.1	1721.2
PUR 20/5 O	3347.3	1721.0			
PUR 30/5	3360.6	1721.2	PUR 20/5+Ch2.5%	3348.7	1720.9
PUR 30/5 H	3341.8	1724.3	PUR 20/5+Ch2.5% H	3370.2	1721.4
PUR 30/5 O	3370.2	1721.0	PUR 20/5+Ch2.5% O	3370.0	1721.1
PUR 0/15	3360.2	1723.7	PUR 20/5+PLA	3352.6	1723.8
PUR 0/15 H	3348.2	1721.3	PUR 20/5+PLA H	3351.4	1721.4
PUR 0/15 O	3341.1	1721.9	PUR 20/5+PLA O	3369.8	1721.2
PUR 10/15	3354.3	1721.5	PUR 20/5+St	3351.9	1720.9
PUR 10/15 H	3340.4	1722.0	PUR 20/5+St H	3343.3	1721.1
PUR 10/15 O	3368.4	1721.3	PUR 20/5+St O	3368.7	1721.0
PUR 20/15	3360.4	1722.8	PUR 20/15+PLA	3366.7	1724.5
PUR 20/15 H	3369.5	1721.9	PUR 20/15+PLA H	3350.5	1721.7
PUR 20/15 O	3354.3	1721.3	PUR 20/15+PLA O	3346.2	1720.5
PUR 15/20	3360.2	1723.3			
PUR 15/20 H	3370.2	1721.3			
PUR 30/20	3316.3	1721.6			
PUR 30/20 H	3350.6	1721.5			
PUR 30/20 O	3356.6	1721.6			
PUR 45/20	3342.6	1722.4			
PUR 45/20 H	3326.0	1721.3			
PUR 45/20 O	3370.4	1723.1			

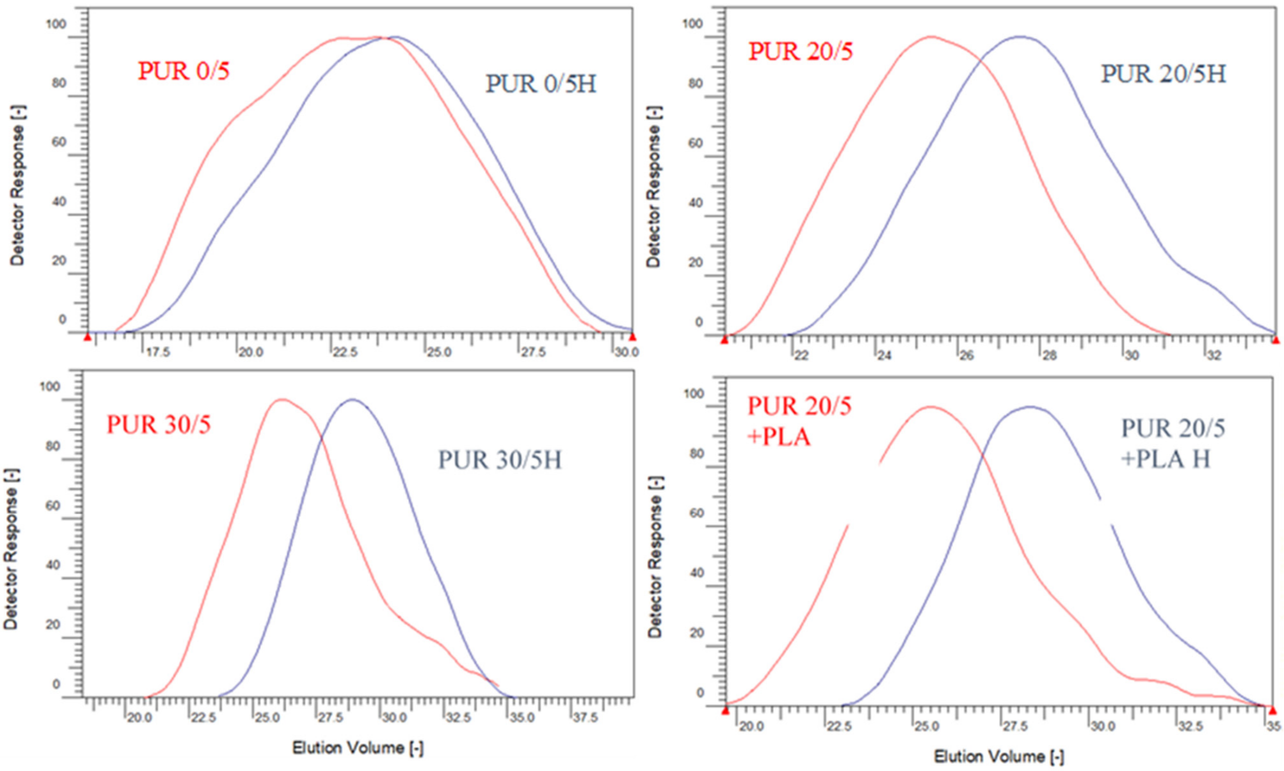

Figure S3. Chromatograms (RI traces) of PUR 0/5, PUR 20/5, PUR 30/5 and PUR 20/5+PLA before (red line) and after (blue line) 36 weeks of incubation in hydrolytic solution (DMF, 1 mL/min)

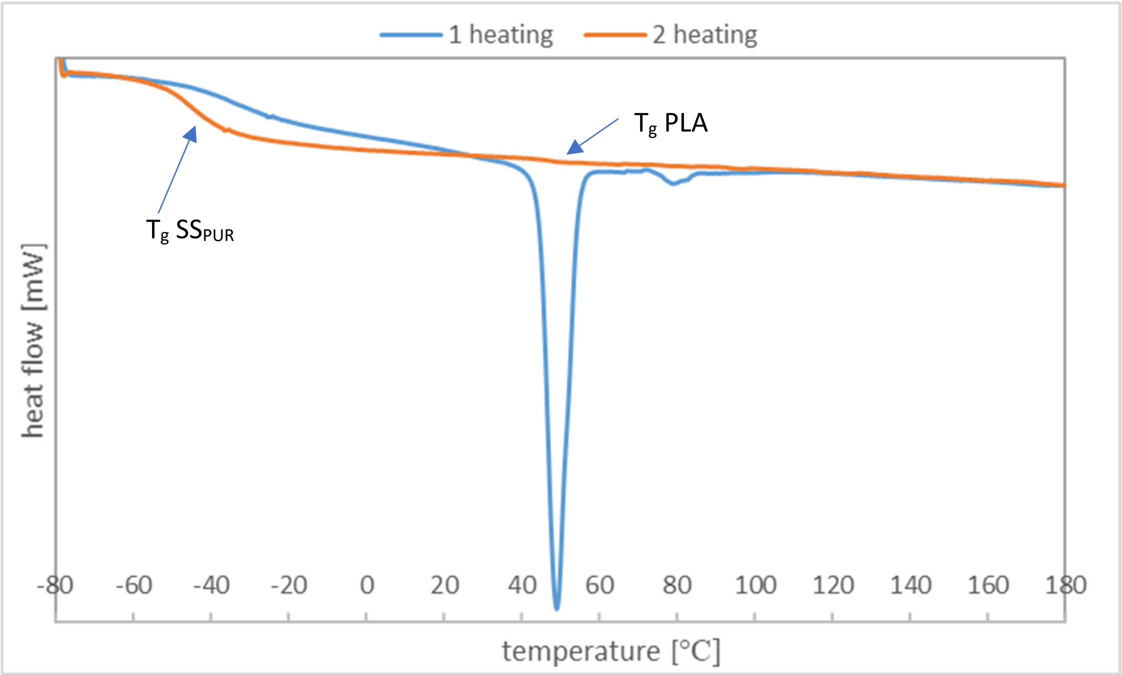

Figure S4. DSC thermogram of PUR 10/5+PLA heated from -80°C to 180°C (1 heating), cooled to -80°C and again heated to 180°C (2 heating)

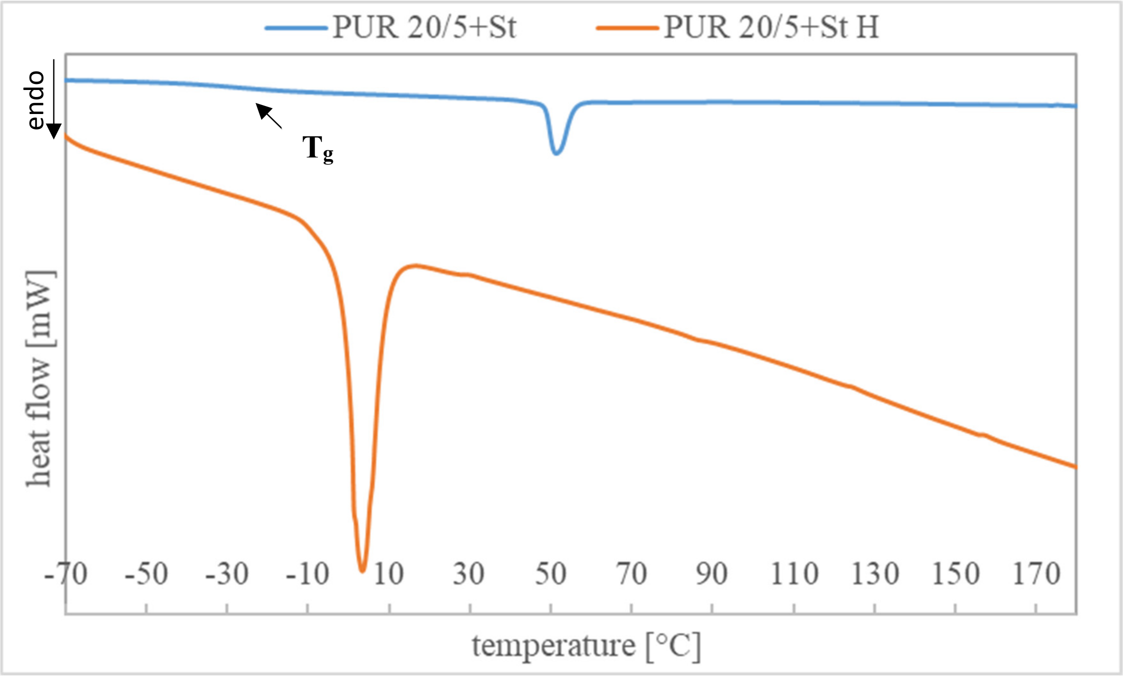

Figure S5. DSC thermogram of PUR 20/5+St before and after incubation in buffer solution

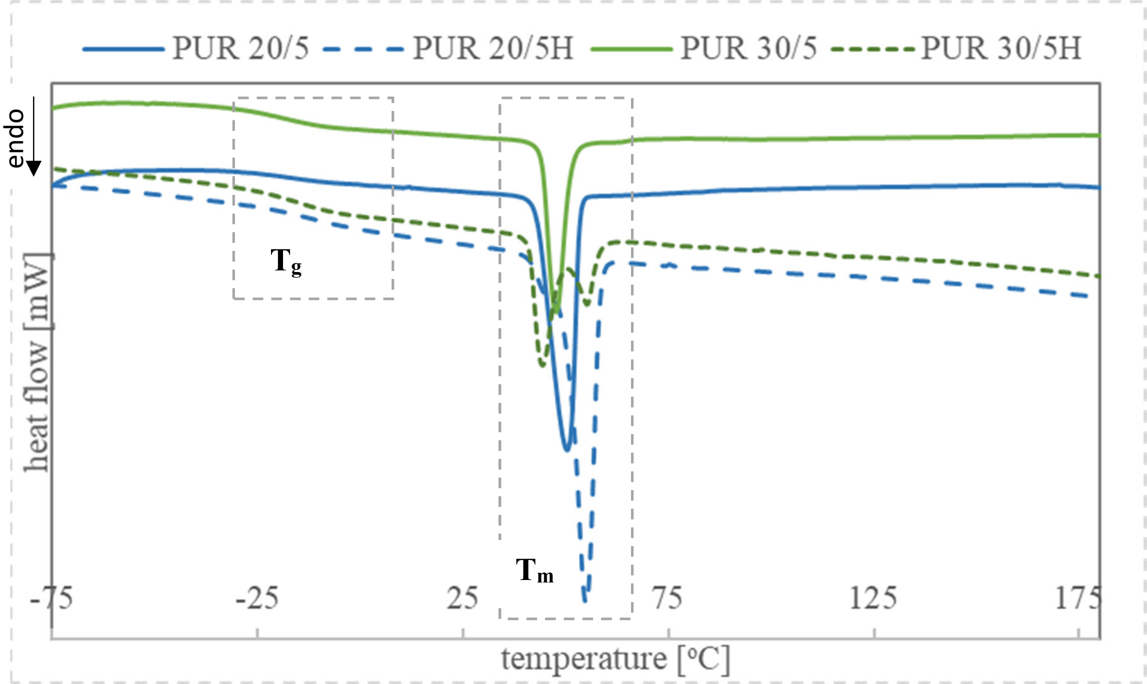

Figure S6. DSC thermogram of PUR 20/5 and PUR 30/5 before and after incubation in buffer solution
